# Left and Right Atrial Strain Across the Heart Failure Continuum: Multifaceted Markers of Subclinical Mechanical Dysfunction and Haemodynamic Burden

**DOI:** 10.3390/diagnostics16182965

**Published:** 2026-09-14

**Authors:** Christos Mantzios, Marino Basha, Sotiria Iliopoulou, Eleftheria Baltagianni, Amalia Baroutidou, Dimitrios Tavoularis, Vasileios Anastasiou, Matthaios Didagelos, Antonios Ziakas, Vasileios Kamperidis

**Affiliations:** 1 1st Department of Cardiology, School of Medicine, Aristotle University of Thessaloniki, AHEPA University Hospital, 54636 Thessaloniki, Greeceb.elira@hotmail.com (E.B.); manthosdid@yahoo.gr (M.D.);; 2 Cardiology Department, G. Papanikolaou General Hospital, 57010 Thessaloniki, Greece

**Keywords:** left atrial strain, right atrial strain, heart failure, subclinical dysfunction, diastolic dysfunction, speckle-tracking echocardiography, atrial remodeling, HFpEF, risk stratification

## Abstract

Heart failure (HF) is increasingly recognized as a progressive syndrome that begins long before patients develop clinical signs or symptoms, during which conventional echocardiographic parameters may fail to detect early myocardial impairment. Atrial strain imaging by speckle-tracking echocardiography has emerged as a sensitive, non-invasive tool capable of identifying subtle functional abnormalities before symptoms or structural remodeling occur. Impaired left atrial (LA) reservoir strain (LASr) serves as a sensitive marker of left ventricular (LV) diastolic dysfunction with elevated filling pressures, exercise intolerance, and HF with preserved ejection fraction, with incremental diagnostic or prognostic value reported in selected studies. Reduced LA strain is also strongly associated with atrial fibrillation (AF), valvular heart disease progression, adverse outcomes following myocardial infarction, and pulmonary vascular involvement. Although comparatively less studied, right atrial (RA) reservoir strain (RASr) reflects the haemodynamic burden of the right heart, right ventricular–pulmonary arterial coupling, and early subclinical dysfunction in cardiometabolic disease, pulmonary hypertension, and established HF. Similarly, RA strain has demonstrated potential diagnostic and prognostic value across the HF spectrum. This review summarizes contemporary evidence regarding the pathophysiological, diagnostic, and prognostic significance of LASr and RASr in unveiling subclinical dysfunction in HF. Both LA and RA strain may provide complementary information on atrioventricular coupling and may identify functional impairment and increased haemodynamic burden before overt structural remodeling becomes evident. Atrial strain assessment may therefore complement conventional echocardiography in the early detection, phenotypic characterization, and risk stratification of patients across different stages of HF.

## 1. Introduction

Heart failure (HF) epidemiology has shifted substantially over the past decades, with heart failure with preserved ejection fraction (HFpEF) now accounting for the majority of all cases and continuing to increase in prevalence [1]. This shift reflects the aging population and its growing burden of comorbidities, including hypertension, obesity, diabetes, and atrial fibrillation (AF) [2]. Importantly, HF is no longer regarded as a purely symptomatic syndrome. Contemporary classifications emphasize earlier stages of the HF continuum, including Stage A, comprising individuals at risk for HF, and Stage B (pre-HF), characterized by structural or functional cardiac abnormalities in the absence of HF symptoms [3,4]. In the present review, the term “subclinical dysfunction” mainly refers to functional atrial abnormalities detectable in individuals at risk for HF (stage A) or with pre-HF (stage B), before overt HF symptoms. As disease progresses to symptomatic HF (Stage C), atrial mechanical impairment may become more pronounced and provide additional information regarding HF phenotype, haemodynamic burden and prognosis. Therefore, selected studies involving patients with established HF are also discussed to illustrate the role of atrial strain across the different stages of HF in detecting atrial dysfunction that may not be apparent by conventional measures.

Elevated LV filling pressures and impaired diastolic relaxation are central pathophysiological features of HFpEF that manifest early in the disease course [5] In this context, left atrial (LA) reservoir strain (LASr), assessed by 2-dimensional speckle-tracking echocardiography (STE) as a measure of myocardial deformation during LA filling, has emerged as a sensitive marker of diastolic dysfunction [6]. It provides unique insight into atrioventricular coupling through its robust correlation with invasively measured LV filling pressures across the full spectrum of ejection fraction [7,8].

In contrast, data regarding right atrial (RA) function remain comparatively limited. The RA plays a crucial role in modulating right ventricular (RV) filling, reflecting not only RV haemodynamic burden but also right-sided systolic and diastolic function [9]. Assessment of RA function, beyond conventional dimensional measurements, using RASr derived by STE has emerged as a promising tool for risk stratification in patients with HF [9].

**Figure 1:**
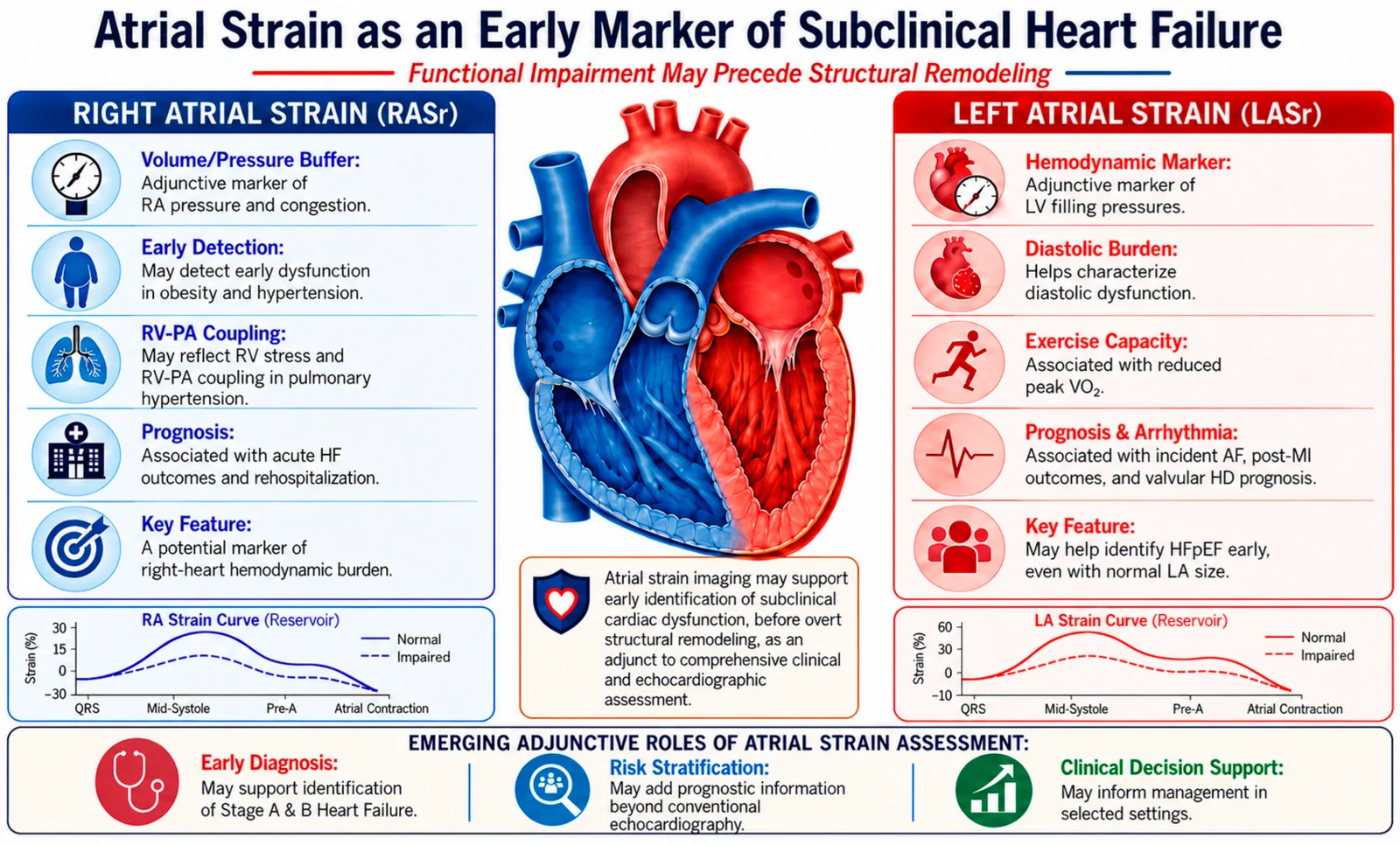
Left and right atrial strain as complementary markers across the heart failure continuum, reflecting distinct aspects of left- and right-sided haemodynamic dysfunction, with potential diagnostic, phenotypic, and prognostic relevance in pre-HF, established HF, and related cardiovascular conditions.

The aim of this review is to summarize contemporary evidence regarding the role of LASr and RASr, assessed by STE, in the detection of early atrial dysfunction and in the haemodynamic and prognostic characterization of patients across the different stages of HF and in cardiovascular conditions predisposing to HF (Figure 1).

## 2. Literature Search Strategy

This narrative review was based on an electronic literature search conducted in PubMed/MEDLINE using the following search terms: (“left atrial strain” OR “left atrial reservoir strain” OR “right atrial strain” OR “right atrial reservoir strain” OR “atrial deformation”) AND (“heart failure” OR “diastolic dysfunction” OR “subclinical dysfunction”). The original literature search was completed up to 15 February 2026, and an updated search was performed on 26 August 2026 during manuscript revision. Relevant references from contemporary guidelines, systematic reviews, meta-analyses, and the reference lists of selected articles were also screened manually. No language restrictions were applied.

Priority was given to original studies evaluating atrial strain in relation to cardiac function, invasive or non-invasive haemodynamic parameters, diagnostic performance, or clinical outcomes. Among original studies, particular consideration was given to larger cohorts, prospective studies, and studies incorporating invasive haemodynamic validation. Systematic reviews, meta-analyses, and contemporary guideline documents were also considered where relevant. Table 1 and Table 2 include representative studies covering the principal clinical settings addressed in this review. No formal predefined inclusion or exclusion criteria or systematic study-selection workflow were applied; studies were selected based on their relevance to the aims of the review. Given the narrative nature of this review, no formal systematic-review protocol or predefined risk-of-bias assessment was applied, and selection bias cannot be excluded.

## 3. Left Atrial Strain as a Marker of Subclinical Dysfunction

### 3.1. Diastolic Dysfunction in Sinus Rhythm

**Figure 2:**
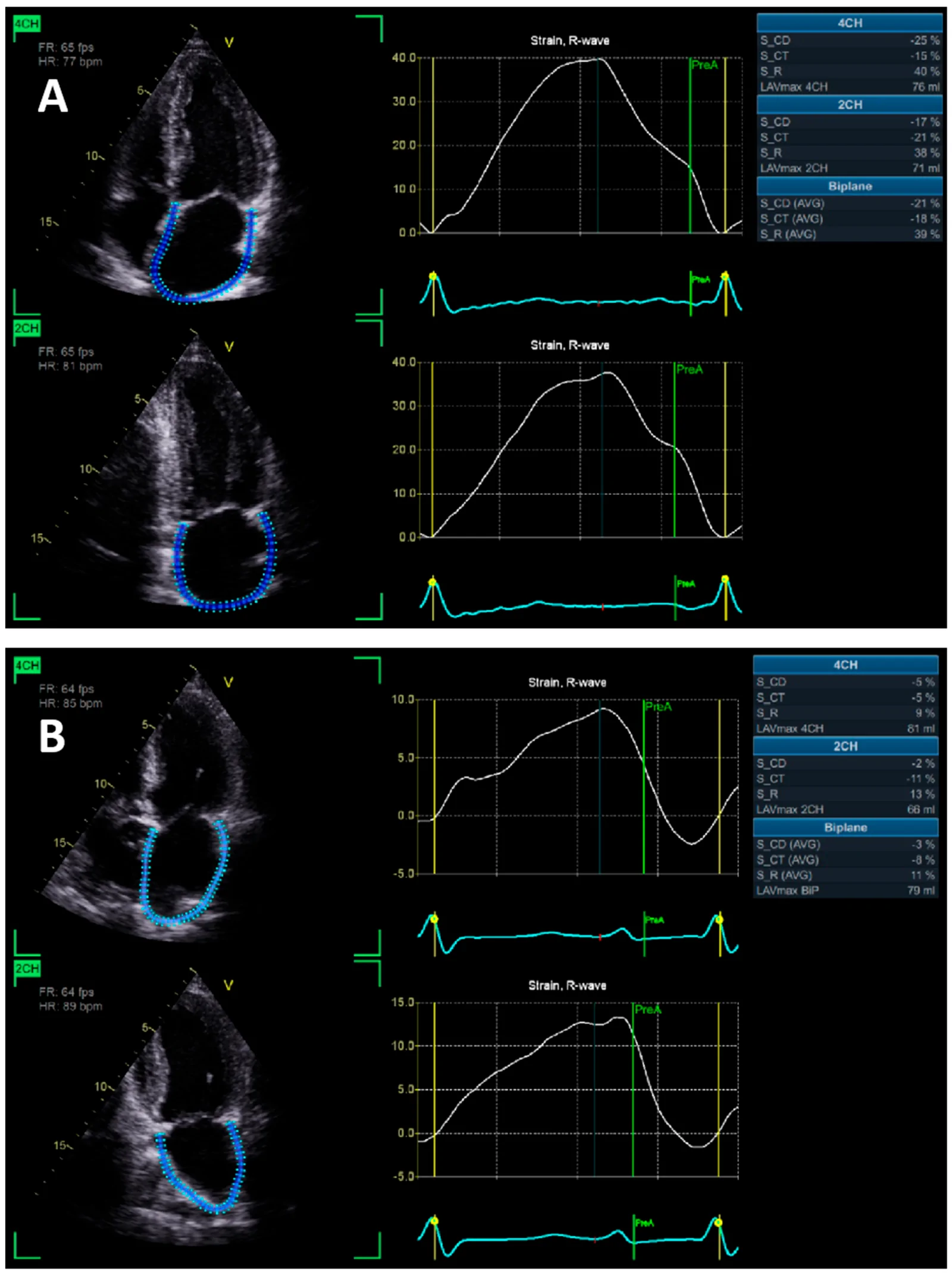
Left atrial strain assessed by speckle-tracking echocardiography: (**A**) Normal left atrial strain demonstrating preserved reservoir, conduit, and contractile phases, with high peak reservoir strain values. (**B**) Impaired left atrial strain characterized by reduced reservoir function and altered strain curve morphology, consistent with elevated left ventricular filling pressures in a patient with HFrEF and severe aortic stenosis. The illustrated strain curves were obtained from fully de-identified echocardiographic examinations of patients evaluated at our institution and are presented for illustrative purposes. No patient-identifying information is displayed.

Throughout the cardiac cycle, the LA functions sequentially as a reservoir during ventricular systole, as a conduit during early diastole, and as an active booster pump during late diastole [25]. Speckle-tracking echocardiography quantifies LA mechanics across these phases by measuring reservoir strain (LASr), conduit strain (LAScd), and contraction strain (LASct) [26], as illustrated by representative normal and pathological strain patterns in Figure 2. Among these three parameters, LASr has been most extensively evaluated for the assessment of diastolic dysfunction in sinus rhythm, either as an additional metric or as a substitute for individual indices within the guideline-proposed algorithm [10,15,27]. These findings are reinforced by biochemical correlations, as LASr exhibits a strong inverse relationship with N-terminal pro-B-type natriuretic peptide (NT-proBNP) (r = −0.75), a widely validated biomarker for the diagnosis and prognostic assessment of HF [28]. Consistent with progressive impairment, LASr demonstrates a stepwise decline across the three grades of diastolic dysfunction, supporting its association with increasing diastolic dysfunction severity [8,10]. Frydas et al. reported that LASr identified severe diastolic dysfunction in patients with HF with an AUC of 0.83 using a study-specific cut-off of 14.1%, outperforming conventional parameters including LAVI and E/e′ [8]. In a separate cohort of patients with preserved LVEF, Singh et al. found that an LASr threshold of 19% discriminated grade 3 diastolic dysfunction from grades 0–2 with an AUC of 0.91 and a diagnostic accuracy of 95% [10]. Beyond its ability to discern advanced disease, LASr is also highly sensitive to early functional impairment that may precede overt atrial enlargement, making it particularly attractive for identifying subclinical disease, unlike LA volume, which reflects chronicity of atrial remodeling. Notably, a LASr cut-off of 23% unveiled abnormal LA function in 60.6% of patients with normal LA volume index [8].

Beyond resting haemodynamics, LA strain has also been associated with exercise capacity [29]. In asymptomatic individuals with HF risk factors, subclinical LV impairment, defined by abnormal LV strain, is associated with significantly reduced peak VO_2_, indicating early exercise intolerance, characteristic of Stage B HF [30]. Similarly, in the study by Reddy et al., an LASr threshold of ≤24.5% showed good discriminatory performance for distinguishing HFpEF from non-cardiac causes of dyspnea [12]. Diagnostic performance is further enhanced by indexing LASr to E/e′, creating a surrogate measure of LA compliance [12]. For those with newly developed symptoms of HF, impaired LASr has been associated with severely reduced exercise capacity, unveiling the association between HF symptoms and myocardial dysfunction [16]. Specifically, in the cohort studied by Maffeis et al., LASr < 23% was associated with 16-fold higher odds of impaired aerobic capacity and remained independently associated with peak VO_2_ across the whole range of ejection fraction [16].

### 3.2. HFpEF and Atrial Fibrillation

Atrial fibrillation (AF) is highly prevalent in HFpEF and may be a predisposing factor to the development of symptomatic HF. In AF, LA volume is often increased, while LASr may serve as a surrogate marker for the assessment of diastolic dysfunction diastolic dysfunction due to rhythm irregularity and loss of atrial contraction [31,32]. LASr retains a role in the guideline-recommended diagnostic algorithm, in case the four primary parameters [E, E/e′, E deceleration time and tricuspid regurgitation (TR) velocity] are inconclusive [31,32]. LASr provides additional diagnostic information by identifying impaired LA function before formal guideline criteria for diastolic dysfunction are fulfilled and by reflecting the increasing burden of echocardiographic abnormalities. In the study by Jarasunas et al., mean LASr decreased from 35.82 ± 6.93% in patients with no diastolic dysfunction parameters to 30.48 ± 6.10% and 26.68 ± 7.68% in those with one and two diastolic dysfunction parameters, respectively [33].

Beyond the assessment of diastolic dysfunction and HF in AF, LASr has also been independently associated with incident AF in sinus rhythm patients; LASr ≤ 18% in patients with HF was independently associated with new-onset AF over a five-year period [13]. Park et al. developed the HAS-BAP score (hypertension, age, LASr, absence of beta-blocker prescription at discharge, atrial volume index, and HFpEF), integrating atrial mechanics with clinical risk factors, to effectively stratify the risk of developing AF [13]. The predictive value of LA strain for AF occurrence extends beyond HF populations. In the Copenhagen City Heart Study, reduced LASr was independently associated with incident AF in community-based individuals, despite normal LA size and preserved LV systolic function, highlighting the ability of LA strain to identify early atrial dysregulation [17]. Furthermore, reduced LASr correlates strongly with histologically confirmed LA fibrosis, reinforcing its role as a non-invasive marker of atrial remodeling and AF susceptibility [34].

Reduced LASr therefore reflects not only haemodynamic burden but also structural atrial remodeling, bridging the pathophysiological gap between diastolic dysfunction and arrhythmogenesis in HF.

### 3.3. Pulmonary Hypertension and Right-Heart Involvement in HFpEF

In HFpEF, pulmonary hypertension due to left heart disease is common and may be accompanied by progressive right-heart involvement, while differentiation from pre-capillary pulmonary hypertension can be clinically challenging [35]. This phenotype arises from chronic retrograde transmission of elevated left-sided filling pressures through the pulmonary circulation, culminating in impaired RV–pulmonary arterial coupling. Given the central role of diastolic dysfunction in HFpEF, precise identification of elevated filling pressures is fundamental to diagnosis [35]. While right heart catheterization remains the haemodynamic gold standard, its invasive nature, limited feasibility, and inability to capture dynamic physiological states constrain widespread application [36]. Consequently, there is growing interest in non-invasive markers capable of integrating left-sided diastolic burden with pulmonary and RV involvement. The 2025 American Society of Echocardiography guidelines incorporate lateral E/e′, mitral E/A ratio, LA volume index, and LASr into the multiparametric assessment of LV filling pressures, with LASr < 18% supporting the presence of elevated filling pressures [9,31]. In patients with pulmonary hypertension, reduced LASr may therefore provide additional evidence of left-sided haemodynamic involvement and, when interpreted alongside conventional echocardiographic parameters and the overall clinical context, rather than as a stand-alone measurement, may contribute to the differentiation of HFpEF-related pulmonary hypertension from pre-capillary disease. In this setting, LA strain may provide complementary information by reflecting the chronicity and severity of LV diastolic dysfunction and upstream pressure transmission to the pulmonary circulation [37]. Reduced LASr has been consistently associated with higher pulmonary artery pressures, increased pulmonary vascular resistance, and worse RV–pulmonary artery coupling [38]. Importantly, LA strain abnormalities may be present even when resting pulmonary pressures are modest, suggesting a role in identifying early or exercise-induced HFpEF phenotypes [39]. These findings highlight LA strain as a key integrative marker linking LV diastolic dysfunction to pulmonary and RV involvement, thereby contributing to early phenotypic characterization in HF.

### 3.4. LA Strain in Valvular Heart Disease: Mitral Regurgitation and Aortic Stenosis

Valvular heart disease is a major predisposing factor to HF, often through chronic pressure or volume overload, and LA strain has emerged as a valuable prognostic marker in this setting, even before symptoms appear. In patients with HF and concomitant mitral regurgitation (MR), LASr provides incremental prognostic value beyond structural and functional parameters, whereas LA volume index does not, further supporting the idea that LA functional impairment precedes structural remodeling [18].

In secondary ventricular MR, LASr exhibits a near-linear relationship with cardiovascular events across its full spectrum, and when reduced, it amplifies the adverse prognostic impact of functional MR, highlighting the contribution of diminished LA functional reserve in worse outcomes [40]. In patients with severe primary MR undergoing mitral valve repair, reduced preoperative LASr has been independently associated with all-cause mortality and has demonstrated incremental prognostic value beyond conventional parameters, although its potential role in guiding the timing of intervention remains to be determined [34,41]. Conversely, improvements in LA strain following interventional repair or pharmacologic therapy are associated with reduced mortality and HF hospitalization, indicating that recovery of atrial mechanics is associated with meaningful reverse remodeling [42].

LA strain has demonstrated prognostic value in patients with aortic stenosis, providing additional information beyond conventional echocardiographic parameters and biomarkers. In asymptomatic patients with moderate AS, reduced LASr was independently associated with adverse cardiovascular events and showed high discriminatory performance for medium-term outcomes, exceeding that of E/e′ and NT-proBNP in this cohort [43,44]. However, whether LASr-guided surveillance or earlier intervention improves clinical outcomes remains to be studied. Additionally, Sabatino et al. found that a decrease or limited improvement in LA strain following transcatheter aortic valve replacement was independently associated with cardiovascular death and HF hospitalization during follow-up, supporting the prognostic relevance of changes in LA strain after TAVI [14].

### 3.5. Prognostic Value After Myocardial Infarction

Myocardial infarction may lead to HF through functional and structural cardiac remodeling. Emerging evidence supports the prognostic relevance of LASr following myocardial infarction. Unlike traditional volumetric assessments, which reflect chronic remodeling, LASr may identify mild diastolic dysfunction and subclinical mechanical impairment before overt geometric changes occur [45,46]. These findings are consistent across imaging modalities, with strong agreement between echocardiography and cardiac magnetic resonance imaging. Large-scale magnetic resonance studies have validated specific LASr thresholds (<18.8%) that provide incremental prognostic value beyond infarct size and LV ejection fraction [11,45].

Clinically, impaired LASr has been associated with both arrhythmic and HF-related outcomes. In patients following acute myocardial infarction, reduced LASr has been independently associated with new-onset AF, with lower strain values associated with an approximately four-fold higher arrhythmic risk [45,47]. Beyond AF, LASr functions as an integrative marker of cardiac performance and has been independently associated with composite endpoints of mortality, reinfarction, and HF hospitalization, with incremental prognostic value demonstrated in selected studies [11,46,48].

Although one study reported attenuation of LASr’s independent prognostic value after adjustment for global longitudinal strain and LA volume index [49], the overall body of evidence suggests that LASr captures distinct pathophysiological information, particularly in the subclinical phase when overt LA enlargement or significant LV impairment has not yet developed [11,45,46,47,48]. Differences in patient populations, timing of assessment, and analytical methodologies may explain this discrepancy.

Taken together, the available evidence demonstrates that assessment of LA strain provides additional information on atrioventricular coupling and may identify functional abnormalities before overt geometric remodeling. Available studies suggest that LA strain may contribute to early phenotypic characterization and risk stratification across several cardiovascular conditions (Table 1).

## 4. Right Atrial Strain as a Marker of Subclinical Dysfunction

**Figure 3:**
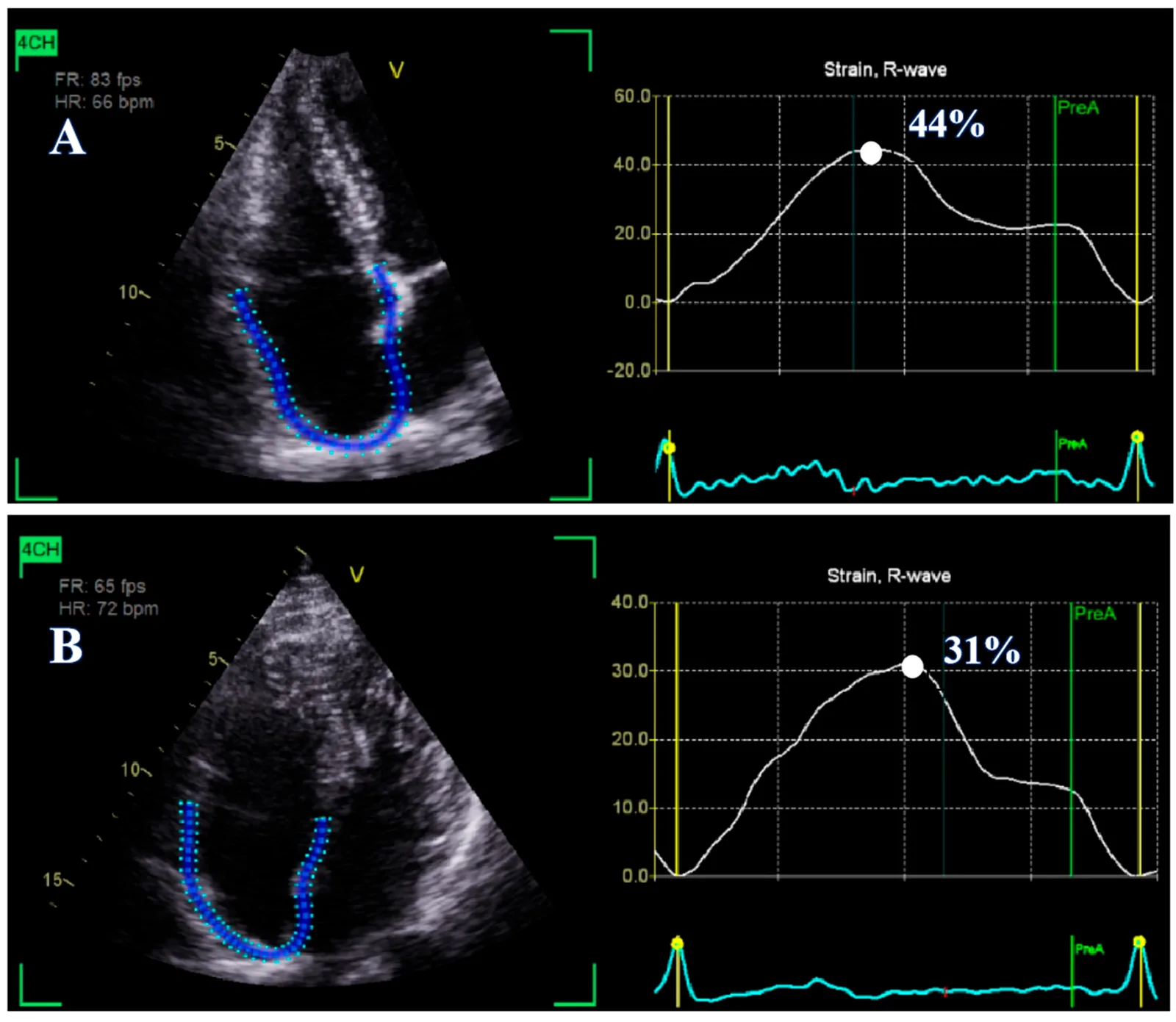
Right atrial reservoir strain assessed by speckle-tracking echocardiography: (**A**) Normal right atrial strain with preserved reservoir function. (**B**) Reduced right atrial strain showing impaired reservoir function and blunted strain curve, reflecting early right-sided dysfunction and altered right ventricular filling dynamics in a patient with coronary artery disease undergoing coronary artery bypass grafting. The illustrated strain curves were obtained from fully de-identified echocardiographic examinations of patients evaluated at our institution and are presented for illustrative purposes. No patient-identifying information is displayed.

The crucial role of RA is to modulate cardiac filling during the systolic and diastolic phases. Acting as a volume buffer, RA may reflect alterations in right-sided haemodynamic conditions in the diagnosis of subclinical heart failure. RA strain impairment evaluated by STE often precedes structural remodeling (Figure 3) [9]. A recently published meta-analysis including 4111 healthy subjects from 21 studies reported an average RASr of 44%; for RASr specifically, the estimated normal range was 25–63%, based on 20 studies including 3921 individuals [50]. Importantly, substantial between-study heterogeneity was observed (I^2^ = 89.3%), highlighting the current variability in reported RASr reference values [50].

### 4.1. Early Cardiovascular Risk States

The role of RA mechanics in early asymptomatic cardiovascular disease has been highlighted in individuals with HF risk factors and pre-HF. Madronio et al. collected measurements of cardiovascular haemodynamics and myocardial mechanics from “healthy” obese individuals [51]. Their analysis demonstrated significantly reduced RASr, assessed by cardiac magnetic resonance, in the individuals with obesity compared with controls (28% vs. 34%, respectively), suggesting early atrial myopathy associated with obesity due to increased cardiac output and stroke volume. Similarly, in a retrospective study enrolling hypertensive patients with preserved LVEF, RASr was significantly impaired compared with normal controls (34.8% ± 13.7% vs. 45.0% ± 13.3%), even in the absence of LV hypertrophy [22]. The authors suggested that this early RA dysfunction may be related to chronic systemic pressure overload, potentially promoting diffuse myocardial remodeling, increased myocardial stiffness, and biventricular fibrotic changes, which may impair atrial compliance and ventricular–atrial coupling before overt structural remodeling becomes apparent. Therefore, these observations suggest that obesity and hypertension, as major HF risk factors, are associated with impaired RASr, which may represent an early subclinical marker of cardiovascular dysfunction in patients at risk for HF.

### 4.2. Established HFpEF and Pulmonary Vascular Disease

RASr was found to be significantly decreased in women at early stages of LV diastolic dysfunction, compared to women with normal diastolic function (35.1 ± 10.4% vs. 43.1 ± 11.9%, respectively; *p* < 0.01) [52,53]. When studying patients with established HFpEF, Kagami et al. observed significantly reduced RASr compared with control subjects (27.0 ± 17.1% vs. 38.6 ± 17.1%, *p* < 0.0001), despite normal RA size [21]. Furthermore, the authors demonstrated correlation between reduced RASr and impaired cardiac output, RV systolic function, and peak oxygen uptake during exercise. From a haemodynamic perspective, in a study of 103 HF patients who underwent right heart catheterization, RASr was independently associated with invasive RA pressure, with a study-specific threshold of ≤15% showing an AUC of 0.78 and higher specificity than IVC-based RAP assessment [20].

Moreover, the assessment of RA strain allows for a better understanding of RV haemodynamics in pulmonary hypertension, since the impaired RASr reflects RV pressure and volume overload [54]. Complementing this, Piccinino et al. demonstrated that RA global strain was independently associated with invasively measured cardiac index and showed stronger associations with haemodynamic impairment than conventional RA size measurements [19].

### 4.3. Prognostic Value Across the Heart Failure Spectrum

Beyond its diagnostic role, RA strain offers significant prognostic value for arrhythmic and acute heart-failure-related complications, as well as mortality. Tomaselli et al. revealed that in AF patients undergoing elective cardioversion, reduced RASr was independently associated with AF recurrence and showed higher discrimination than LASr [23]. These findings suggest that impaired RASr may reflect additional aspects of functional and electrical atrial remodeling. In patients hospitalized with acute HF, Anastasiou et al. showed that impaired RASr < 11.4% at discharge was independently associated with mortality and HF rehospitalization and provided incremental prognostic value beyond RA size and RV structural and functional remodeling [24]. These findings indicate that RA dysfunction is not simply a transient haemodynamic parameter, but a marker of vulnerability that manifests early in the subclinical phase and extends across the entire spectrum of HF.

Taken together, the available evidence indicates that assessment of RA strain provides additional information about functional remodeling, often detecting abnormalities before structural changes. As highlighted by the studies presented in Table 2, its diagnostic and prognostic associations suggest that RA strain may serve as a complementary marker for early phenotypic characterization and risk stratification, although its role in guiding clinical management remains to be established. However, compared with LASr, the evidence supporting RASr remains more limited and is largely derived from smaller or selected cohorts; therefore, its clinical role requires further validation in larger prospective studies.

## 5. Complementary Roles of Left and Right Atrial Strain

LASr is an imaging-derived estimation of LA mechanics and function that is associated with LV mechanical function and filling pressures [55]. This interdependence reflects the LA-LV coupling, as LV longitudinal shortening contributes to LA reservoir deformation through displacement of the mitral annulus. A recent CMR study further supports this interaction, showing a close association of LASr with both LV global longitudinal strain and atrioventricular plane displacement [56]. Therefore, impaired LASr may reflect not only intrinsic LA dysfunction but also abnormalities in LV longitudinal mechanics and loading conditions [55,56]. Respectively, RASr has been associated with right-sided haemodynamics and ventricular abnormalities [19,20]. In patients with HF, RASr has been independently associated with invasively measured RA pressure, and has also been related to cardiac index and pulmonary vascular resistance in pulmonary hypertension [19,20]. Moreover, exercise studies in HFpEF have linked impaired RA strain reserve with reduced RV systolic reserve, cardiac output, and exercise capacity [21]. Taken together, LASr and RASr may provide different, although corresponding and interdependent, information regarding left- and right-sided atrioventricular coupling and haemodynamic burden.

Beyond these associations, recent studies indicate that simultaneous assessment of LASr and RASr may provide complementary information. In patients with persistent AF undergoing electrical cardioversion, Tomaselli et al. showed that both LASr and RASr were associated with AF recurrence, while RASr showed higher discrimination and provided incremental prognostic information beyond LASr [23]. In acute HF, Anastasiou et al. demonstrated that RASr provided additional prognostic information beyond a model that already included LASr [24]. More recently, Nakayama et al. evaluated LASr and RASr simultaneously in 1147 patients with chronic HF and LVEF < 50% and found that concomitant impairment of both parameters identified a subgroup with the highest risk of cardiac death or HF hospitalization [57].

These findings suggest that bi-atrial reservoir strain assessment may provide complementary information by integrating abnormalities across both sides of the heart, providing insight to the global cardiac mechanics with hemodynamic implications. However, the available evidence remains largely observational and derives from selected clinical populations; therefore, the incremental clinical value of routinely combining LASr and RASr assessment, and whether it can improve clinical decision-making or outcomes, requires further prospective validation.

## 6. Technical Considerations, Limitations and Future Directions

The accurate assessment of atrial strain depends strongly on appropriate image acquisition and analysis. Apical views should be obtained without foreshortening and with adequate visualization of the atrial walls, while image depth, sector width, and frame rate must be optimized to achieve sufficient spatial and temporal resolution [26,58]. Given that atrial walls are thin, suboptimal image quality and acoustic dropouts may affect myocardial tracking, reducing measurement feasibility and reproducibility [26,59]. Therefore, LA-focused and RA-focused apical views are recommended for LASr and RASr assessment, respectively, because the atrial long axes may differ from those of the corresponding ventricles [58]. Image depth and sector width should be optimized to achieve a stable frame rate > 50 frames/s throughout atrial acquisition. In case of tachycardia, a higher frame rate may be required to avoid underestimation of strain.

ECG-gated acquisition is also important, as atrial strain values depend on the chosen zero-reference point. Using ventricular end-diastole as the zero-reference point, reservoir strain corresponds to peak positive atrial deformation during ventricular systole, whereas conduit and contraction strain reflect the subsequent changes in atrial deformation during early and late diastole, respectively [26,58]. R–R gating, with ventricular end-diastole as the zero-reference point, is generally recommended because this approach can be applied in both sinus rhythm and AF, whereas gating based on the onset of atrial contraction is not applicable in AF [26,58]. Cardiac rhythm represents an additional source of variability. Specifically, the absence of organized atrial contraction in AF and beat-to-beat variation in cycle length and loading conditions complicate strain assessment [31,58]. Therefore, measurements from at least three consecutive cardiac cycles should be averaged in AF, whereas atrial contraction strain cannot be assessed in the absence of organized atrial contraction [58].

Besides heart rhythm and heart rate, atrial strain is influenced by age, sex, atrial size, loading conditions, ventricular function, valvular disease, pulmonary pressure, and treatment status; therefore, reduced LASr or RASr should be interpreted within the clinical context and not as evidence of isolated or primary atrial myopathy alone [31,55,59]. Since most available evidence is observational, residual confounding and reverse causation cannot be excluded. Further prospective multicenter studies using standardized protocols and externally validated reference ranges are needed to determine the incremental clinical value of atrial strain and whether strain-guided assessment can improve clinical outcomes.

Finally, differences among vendors, software packages and modalities remain an important source of measurement variability [26,58]. Strain values obtained by STE and CMR feature tracking should not be considered directly interchangeable, while the use of dedicated atrial-analysis software, when available, may improve methodological consistency compared with ventricular-analysis packages [50,58,59]. Although standardization efforts have improved the consistency of left atrial strain imaging, reference values and clinically significant thresholds may still vary depending on acquisition methodology, software, cardiac rhythm, loading conditions, and the study population [26,50,58,59]. Intra- and interobserver variability may also increase with suboptimal image quality and differences in analysis methodology.

These limitations are particularly relevant for RASr, for which available data and validated reference values remain less extensive compared with LASr [9,50]. Importantly, the different LASr and RASr cut-off values reported in the literature have been derived from heterogeneous populations and refer to different clinical endpoints, including diastolic dysfunction, elevated filling pressures, HFpEF diagnosis, exercise limitation, arrhythmic events, and prognosis. Consequently, the reported atrial strain cut-off values should not be interpreted as universal thresholds, but rather within the specific clinical and methodological context studied. Additional areas requiring further investigation include automated or AI-assisted strain analysis, reference values across diverse demographic populations, and the incremental value of atrial strain beyond established ventricular parameters and HF risk models.

## 7. Conclusions

Atrial strain imaging by STE provides a sensitive, non-invasive assessment of atrial mechanics that may enable the detection of subclinical functional impairment in HF stage A and B and reveal otherwise unrecognized impairment across the diverse clinical phenotypes of symptomatic HF. Evidence for the clinical use of LASr is more explicit, particularly regarding its association with LV diastolic dysfunction and elevated filling pressures, whereas RASr remains a promising complementary marker of right-sided haemodynamic burden and prognosis that requires further standardization and validation. However, differences in acquisition methodology, loading conditions, rhythm, software, and study populations currently limit the use of universal reference values and thresholds, particularly for RASr. Atrial strain should therefore be considered as a complementary echocardiographic parameter rather than a stand-alone diagnostic or prognostic tool. Further prospective multicenter studies are needed to determine its incremental clinical value and whether strain-guided assessment can improve patient management and outcomes.

## Figures and Tables

**Table 1:** Representative studies demonstrating the diagnostic and prognostic role of Left Atrial Strain (LASr) in detecting subclinical dysfunction.

1st Author/ Year	Study Design/Population (*n*)	Imaging/Rhythm	Key LA Findings/Reference Standard	Outcomes/Adjusted or Incremental Evidence/Follow-Up	Main Limitations
Singh et al. [10] 2017	Derivation and validation cohorts; 229 preserved LVEF patients (90 derivation; 139 validation)	2D STE; EchoInsight (Epsilon); sinus rhythm	Study-specific LASr > 24% differentiated normal/grade 1 from grades 2/3 DD	-Good discrimination of diastolic dysfunction (AUC 0.86–0.91; diagnostic accuracy up to 95%)	Single-center retrospective study; selected population; no gold-standard reference for DD.
Schuster et al. [11] 2019	Secondary analysis of two prospective multicenter AMI trials; 1046 analyzable patients with AMI (1235 enrolled)	CMR feature tracking, TomTec Cardiac Performance Analysis; mixed rhythm	Study-derived LASr < 18.8%	-Independently associated with 12-month MACE (adjusted HR 0.95, *p* = 0.02) -Ιncremental value over LVEF & infarct size (AUC comparisons *p* < 0.04)	CMR rather than STE; relatively low event rate; sicker patients unsuitable for CMR excluded
Reddy et al. [12] 2019	Consecutive patients with unexplained exertional dyspnea; 238 HFpEF + 125 NCD	2D STE, Syngo (Siemens); QRS gating; mixed rhythm	Study-derived LASr threshold 24.5%: AUC 0.719, sensitivity 56%, specificity 94%	-Discriminated HFpEF from NCD (AUC 0.72; invasive PCWP reference). -LASr/E/e′ improved diagnostic performance (AUC 0.77; *p* = 0.003). -Cross-sectional validation.	Non-simultaneous echo/RHC; study-specific cut-off not externally validated; incomplete NT-proBNP data.
Frydas et al. [8] 2020	Post hoc analysis of SOCRATES-PRESERVED/REDUCED; 300 HF patients	2D STE, TomTec 2D Cardiac Performance Analysis v4.6; sinus rhythm	LASr ≤ 23% abnormal; cut-off 14.1% for severe DD	-High diagnostic accuracy for severe DD (AUC 0.83); sensitivity 80%, specificity 77.8%; superior to LAVI, LAEF and E/e′	Post hoc analysis; no invasive reference standard; only patients with sinus rhythm and adequate image quality included
Park et al. [13] 2020	Retrospective multi-center acute-HF registry; 2461 HF patients with sinus rhythm	2D STE, Image-Arena v4.6 (TomTec); R–R gating	Study-derived LASr ≤ 18%	-Independently associated with new-onset AF (HR 1.60; 5-year follow-up).	Retrospective design; possible AF underdetection/detection bias; East Asian acute-HF population; no external validation
Sabatino et al. [14] 2021	Single-center observational cohort; 100 patients with severe AS undergoing TAVI	2D STE, EchoPAC v112.99 (GE); sinus rhythm; AF at examination excluded	Impaired LASr after TAVI (no fixed cut-off)	-Independently associated with CV death and HF hospitalization post-TAVI (reported HR 0.80, *p* < 0.001); median follow-up 31 months	Single-center, relatively small cohort; image-quality dependent, exclusion of patients with poor acoustic window
Venkateshvaran et al. [15] 2022	Retrospective dual-center haemodynamic cohort; 210 LVEF ≥ 50% patients with unexplained dyspnea	2D STE, EchoPAC v11 (GE); sinus rhythm	Reduced LASr; AUC 0.76 for PCWP ≥ 15 mmHg; <18% yielded highest accuracy.	-LASr identified elevated PCWP (AUC 0.76). -Improved algorithm accuracy when replacing TR velocity (68%→81%; AUC 0.69→0.77, *p* = 0.001)	Selected cohort with low prevalence of elevated PCWP; pulmonary disease included; no core-lab analysis or external validation
Maffeis et al. [16] 2022	Two-center observational cohort; 171 HF patients	2D STE; TomTec or EchoPAC according to center; sinus rhythm and AF included	LASr < 23%	-Independently associated with reduced exercise capacity (OR 16.0; AUC 0.73); no prognostic follow-up.	Two vendors/software platforms; no control group and no established LASr cut-off; lack of prognostic data.
Hauser et al. [17] 2022	Prospective population-based cohort; 3590 participants analyzed from 4466 individuals initially enrolled	2D STE, Vivid 9/EchoPAC v202 (GE); prevalent AF excluded	Reduced LASr; no single outcome-derived clinical cut-off	-Independently associated with incident AF, even with normal LA size (HR 1.05 per 1% decrease; median follow-up 5.3 years)	719 participants excluded (inadequate image quality); registry-based detection may underestimate asymptomatic AF
Stassen et al. [18] 2022	Retrospective single-center cohort; 666 HF patients with secondary MR	2D STE, EchoPAC v203 (GE); mixed rhythm (including AF)	Median LASr 9.8% used for stratification—not a validated clinical cut-off	-Independently associated with all-cause mortality (HR 0.50; median follow-up 5 years). -Incremental prognostic value beyond LA volume and LV GLS (Δχ^2^ 11.1; *p* < 0.001).	Retrospective single-center design; vendor-specific measurements; HF hospitalization and cause-specific mortality unavailable

Abbreviations: AF: atrial fibrillation; AMI: acute myocardial infarction; AS: aortic stenosis; AUC: area under the curve; CMR: cardiac magnetic resonance; CV: cardiovascular; DD: diastolic dysfunction; HF: heart failure; HFpEF: heart failure with preserved ejection fraction; LA: left atrial; LASr: left atrial reservoir strain; LVEF: left ventricular ejection fraction; LV GLS: left ventricular global longitudinal strain; MACE: major adverse cardiovascular events; MR: mitral regurgitation; NCD: non-cardiac dyspnea; PCWP: pulmonary capillary wedge pressure; TAVI: transcatheter aortic valve implantation.

**Table 2:** Representative studies demonstrating the diagnostic and prognostic role of Right Atrial Strain in detecting subclinical dysfunction.

1st Author/ Year	Study Design/Population (*n*)	Imaging/Rhythm	Key RA Findings/Reference Standard	Outcomes/Adjusted or Incremental Evidence/Follow-Up	Main Limitations
Piccinino et al. [19] 2017	Prospective study; 36 PH vs. 16 control subjects	2D STE (GE/EchoPAC BT11); sinus rhythm; QRS gating	RA global integral strain 11.40 ± 5.22% vs. 25.72 ± 5.95%; 17% ROC threshold	-Independently associated with cardiac index, superior to RA volume/area; AUC 0.83 (17% threshold for preserved cardiac index). -Stronger association than RA volume; no longitudinal follow-up.	Small cohort; sinus-rhythm only; no serial or long-term outcome assessment.
Miah et al. [20] 2021	Cross-sectional haemodynamic study; 103 HF subjects undergoing RHC	2D STE (GE/EchoPAC); sinus rhythm	Study-specific RASr cut-off ≤15% for invasive RAP > 7 mmHg	-Independently associated with invasive RAP; AUC 0.78. -Higher specificity than IVC-based RAP assessment (72% vs. 32%).	Single-center, selected HF cohort; sinus rhythm only; no external validation.
Kagami et al. [21] 2022	Retrospective study; 89 HFpEF vs. 108 control subjects	2D STE during bicycle exercise; rhythm not specifically reported	Reduced resting RASr (27.0 ± 17.1% vs. 38.6 ± 17.1%)	-RA strain during exercise differentiates HFpEF from controls better than resting parameters.	Retrospective single-center study; selected dyspnea population; limited invasive validation; exercise strain dependent on image quality.
Huang et al. [22] 2022	Retrospective case–control study; 126 patients with hypertension vs. 83 control subjects	2D STE (GE Vivid E9/EchoPAC v203); sinus rhythm	Reduced mean RASr in hypertension (34.8 ± 13.7% vs. 45.0 ± 13.3%); no study-specific cut-off	-Reduced RASr also observed before LV hypertrophy; -Cross-sectional analysis.	Retrospective single-center case–control study; selected hypertensive population; no longitudinal follow-up.
Tomaselli et al. [23] 2023	Retrospective single-center cohort; 132 patients with persistent AF undergoing elective ECV	2D STE (GE/EchoPAC); AF at baseline; ≥5 cycles averaged; R-wave gating	Study-specific RASr ≤ 15% for AF recurrence after ECV	-Independently associated with AF recurrence (HR 3.26; 12-month follow-up) -Higher discrimination than Left Atrial Strain (AUC 0.77 vs. 0.69) and incremental prognostic value (*p* < 0.001).	Single-center retrospective study; selected small cohort; no continuous AF monitoring; LA-specific software used for RASr.
Anastasiou et al. [24] 2025	Prospective single-center cohort; 401 acute HF patients	2D STE (GE/EchoPAC); sinus rhythm and AF; QRS gating	Study-derived RASr cut-off <11.4%	-Independently associated with mortality/rehospitalization (HR 1.80; median follow-up 6 months). -Incremental value over RV function & RA size.	Single-center, relatively small cohort; vendor-dependent RASr cut-off; clinically assessed euvolemia.

Abbreviations: AF: atrial fibrillation; AUC: area under the curve; HFpEF: heart failure with preserved ejection fraction; IVC: inferior vena cava; PH: pulmonary hypertension; RA: right atrial; RAP: right atrial pressure; RHC: right heart catheterization; RV: right ventricular.

## Data Availability

No new data were created or analyzed in this study. Data sharing is not applicable to this article.

## Abbreviations

The following abbreviations are used in this manuscript:

AF	Atrial fibrillation
EF	Ejection fraction
HF	Heart failure
HFpEF	Heart failure with preserved ejection fraction
HFrEF	Heart failure with reduced ejection fraction
LA	Left atrial/left atrium
LAScd	Left atrial conduit strain
LASct	Left atrial contraction strain
LASr	Left atrial reservoir strain
LV	Left ventricular/left ventricle
MR	Mitral regurgitation
NT-proBNP	N-terminal pro-B-type natriuretic peptide
PH	Pulmonary hypertension
RA	Right atrial/right atrium
RASr	Right atrial reservoir strain
RV	Right ventricular/right ventricle
STE	Speckle-tracking echocardiography
TR	Tricuspid regurgitation
